# Influence of aging on the relation between head control and hip joint kinematics during crossover stepping

**DOI:** 10.1371/journal.pone.0299850

**Published:** 2024-05-24

**Authors:** Yahiko Takeuchi, Kimiya Fujio

**Affiliations:** 1 Department of Physical Therapy, Josai International University, Chiba, Japan; 2 Department of Rehabilitation for Movement Functions, Research Institute of National Rehabilitation Center for Persons with Disabilities, Saitama, Japan; UFPE: Universidade Federal de Pernambuco, BRAZIL

## Abstract

Falls in older individuals are a serious health issue in super-aged societies. The stepping reaction is an important postural strategy for preventing falls. This study aimed to reveal the characteristics of lateral stepping in response to mechanical disturbance by means of an analysis of the hip joint kinematics in the stepping leg and head stability during crossover steps. The participants included 11 healthy older and 13 younger individuals. An electromagnet-controlled disturbance-loading device induced crossover steps due to lateral disturbance. Responses were measured using a motion capture system and force plates. The righting reaction of the head was quantified by lateral displacement (sway), neck joint kinematics (angle displacement, angular velocity), and neck joint moment during crossover stepping. Moreover, the relationship between the neck lateral bending moment and angular velocity of hip flexion/adduction of the stepping leg was examined. The lateral head sway was significantly larger in the older participants (1.13±0.7 m/s^2^) than in the younger individuals (0.54±0.3 m/s^2^); whereas, the angle displacement (older -14.1±7.1 degree, young -8.3±4.5 degree) and angular velocity (older 9.9±6.6 degree/s, 41.2±27.7 degree/s) of the head were significantly lower in the older than in the younger participants. In both groups, the moment of neck lateral bending exhibited a significant negative correlation with the hip flexion angular velocity of the stepping leg. Correlation analysis also showed a significant negative correlation between the neck lateral bending moment and hip adduction angular velocity only in the older group (r = 0.71, p<0.01). In conclusion, older individuals increased instability in the lateral direction of the head and decreased righting angle displacement and angular velocity of the head during crossover steps. The correlation between neck moment and hip flexion/adduction angular velocity suggested a decrease in step speed due to increased neck muscle tone, which could be influenced by vestibulospinal reflexes.

## Introduction

Falls in older individuals can lead to serious complications, and post-fall syndromes can further reduce activities of daily living and limit social participation. In postural control strategies [1] for older individuals with reduced standing balance, the stepping reaction is the most effective in preventing falls. The stepping reaction provides better mechanical stability by expanding the base of support compared with a feet-in-place reaction that attempts to correct the body alignment around the ankle joint or hip joint [2]. Falls in older individuals are associated with postural instability in the lateral direction [3] and result in a hard impact on the greater trochanter, which is a cause of femoral fractures [4–6]. Thus, there is a strong need to better understand the stepping reactions in the lateral direction.

Three stepping patterns can expand the support base to the lateral side: a crossover step, side-step sequence, and loaded leg step [7]. The crossover step is often used by young adults to cope with lateral disturbances. However, it is difficult for older individuals to control a crossover step because it requires accurate movement of the step foot to avoid contact with the supporting leg and maintain posture with one leg for a long period [8]. Thus, it is crucial to focus on the crossover step to understand the recovery of balance in the lateral direction and its difficulty with aging.

In addition, the stepping reaction involves a forward shift of the center of mass (COM) of the entire body by stepping to expand the base of support. In the crossover step, the ankle or hip joint of the supporting leg becomes the center of rotation. Since the most distal center of rotation is the head, the important receptors are located there, such as hair cells that sense rotational acceleration in semicircular canals and linear acceleration in the utricle and saccule due to otolith movements. Vestibular input is activated by changes in head tilt and induces changes in the postural tone in the neck and extremities [9] as the vestibulocervical or vestibulospinal reflex.

When losing standing balance, a righting reaction occurs to keep the head vertical in space and induce trunk responses for stepping of the lower limbs. To understand the mechanism of balance recovery by crossover stepping, it is important to investigate how the head COM is controlled and positioned in space during the stepping movement. In particular, the head movement relative to the trunk position is essential to evoke vestibulocervical and vestibulospinal reflexes. Previous studies investigating the relationship between head kinematics and step latency in the step reaction have reported that head acceleration in the sagittal plane is significantly greater in older adults than in younger adults [10]; moreover, they found a significant positive correlation between angular velocity and step latency [11]. However, these studies focused on forward and backward step responses and did not investigate head control during lateral step responses, which are associated with falls in older adults. In addition, it would be necessary to investigate the angle and angular velocity of the head righting in space, which may be involved in vestibulocervical and vestibulospinal reflexes, and the relationship between the lateral flexion moment of the neck, which reflects the muscle tension around the head and neck, and the step leg kinematics.

This study aimed to clarify the head control ability of older individuals based on the amount of head swaying, the righting angle displacement, angular velocity, and the lateral neck moments during the crossover step. In addition, we investigated the relationship between the factors involved in head control and the angular velocity of flexion and adduction of the stepping leg hip joint and the kinematic factors of crossover steps.

## Materials and methods

### Participants

Eleven healthy older individuals (3 females, age: 73.3±3.8 years, height: 1.61±6.6 m, weight: 63.5±12.1 kg, body mass index: 24.1±4.1) living in the community were enrolled as study participants, and 13 healthy young individuals (10 females, age: 20.3±0.6 years, height: 1.61±0.1 m, weight: 51.4±6.6 kg, body mass index: 19.8±1.5) were selected as the control group. All 24 participants did not have any known major orthopedic or neuromuscular disease, vestibular disease, or physical disability. Participants were included in the study if they could perform a crossover step in response to lateral disturbance while standing.

The recruitment period for this study was from May 1, 2018 to August 31, 2018.　All participants provided written informed consent after receiving an explanation about the purpose of the experiment in writing and orally. This study was approved by the Ethical Review Committee of Chiba Prefectural University of Health Sciences (2017–034). All experiments were performed in accordance with the guidelines of the committee and with the Declaration of Helsinki.

### Lateral direction disturbance loading

**Fig 1:**
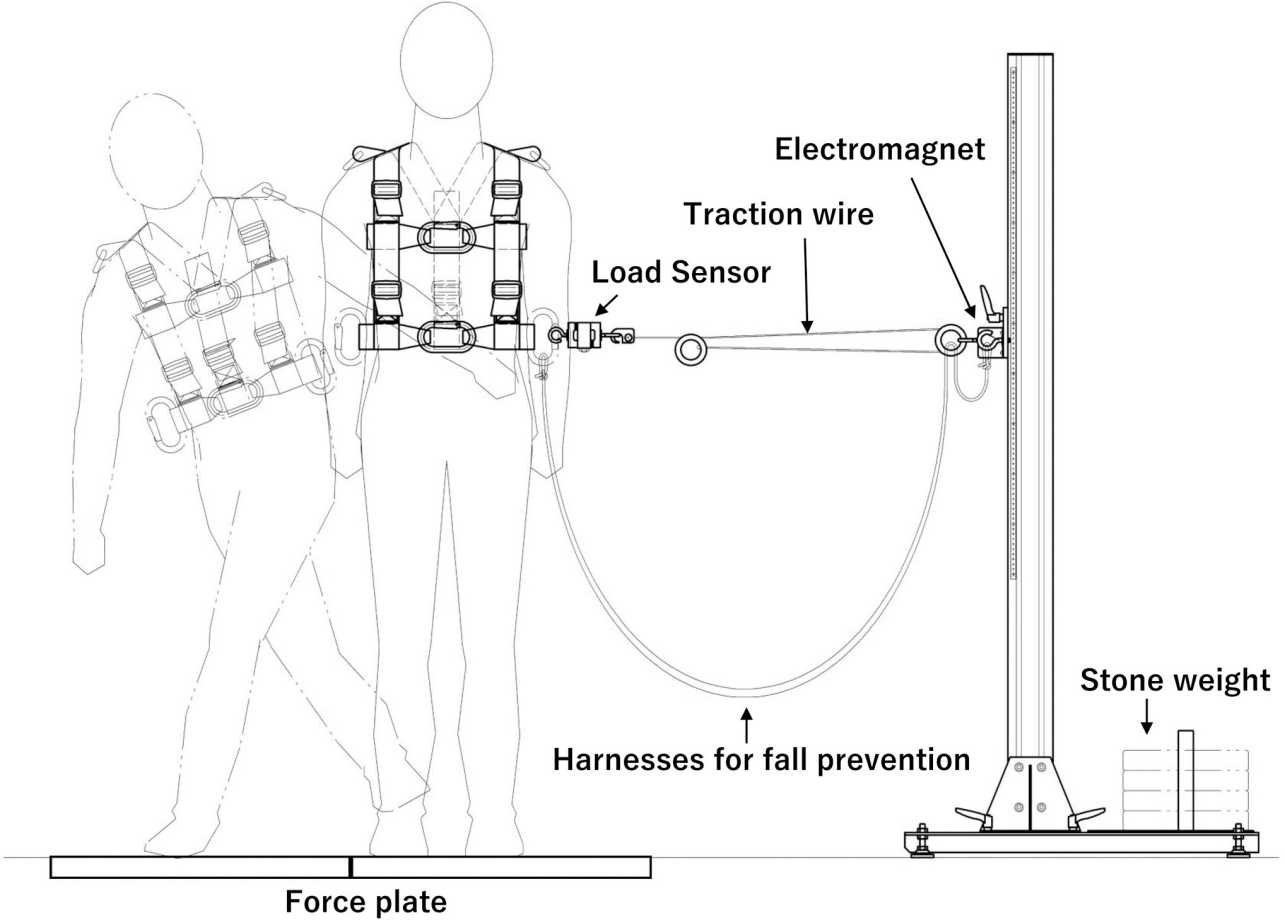
Originally developed disturbance-loading system.

Fig 1 shows an overview of the disturbance-loading device. An electromagnetically controlled standing disturbance-loading device (S-17164, Takei Kiki Kogyo Co., Ltd.) was used for the lateral direction disturbance loading. This device was connected to a load sensor worn by the participant, and a traction wire was connected to the electromagnets. When the traction load exceeded an arbitrary setting, the electromagnets were demagnetized, and the traction wire was disconnected from the electromagnets to cause a disturbance.

The participant maintained a standing posture with equal weight loading on the right and left feet, each positioned on one of two rows of force plates (AMTI, BP400600), with the medial edges of both sides 15 cm apart. The participants were instructed to gaze at an index 2 m in front of them. From this posture, the COM was moved voluntarily to the left. Disturbance was applied to the participant by disengaging the wire from the electromagnet when 10% of the participant’s body weight was applied to the load sensor from the traction wire (Fig 1). We used 10% of the participant’s body weight as the trigger because prior trials have shown that 10% of the body weight was the amount of load at which the lateral step response appeared while ensuring the safety of the sideways fall. Note that participants were not signaled when to load the disturbance to prevent predictive readiness states. The length of the traction wire was standardized among the participants so that the tilt angle displacement of the participant’s body when the disturbance was applied was standardized as much as possible. In addition, after confirming in a preliminary experiment that the participants were able to perform the crossover step with either the left or right leg, we instructed them to perform the crossover stepping with the right leg during the task movement to standardize the measurement conditions. On the other hand, to observe natural responses, participants were not instructed to position their heads during the task movement.

The number of trials in this experiment was set to one to prevent the learning effect from being influenced by repetition.

### Measurement device

The lateral direction step reaction was measured using a three-dimensional motion analyzer (Motion Analysis, Mac3D system) consisting of eight infrared cameras and a force plate. Based on the Helen Hayes marker set [10], 19-mm-diameter infrared reflective markers were placed on 25 points in the participants’ bodies. The sampling frequencies for both the infrared camera and the force plate were set at 100 Hz. Signals from the infrared camera, force plate, and disturbance loader were synchronized via an analog/digital converter connected to a personal computer during data acquisition.

### Data analysis

We analyzed the data from the onset of lateral disturbance to the landing of the stepping leg. The onset of disturbance was identified by a signal from the disturbance-loading device, and the timing of the landing of the stepping leg was identified from the reaction force in the vertical direction measured by the force plate.

**Fig 2:**
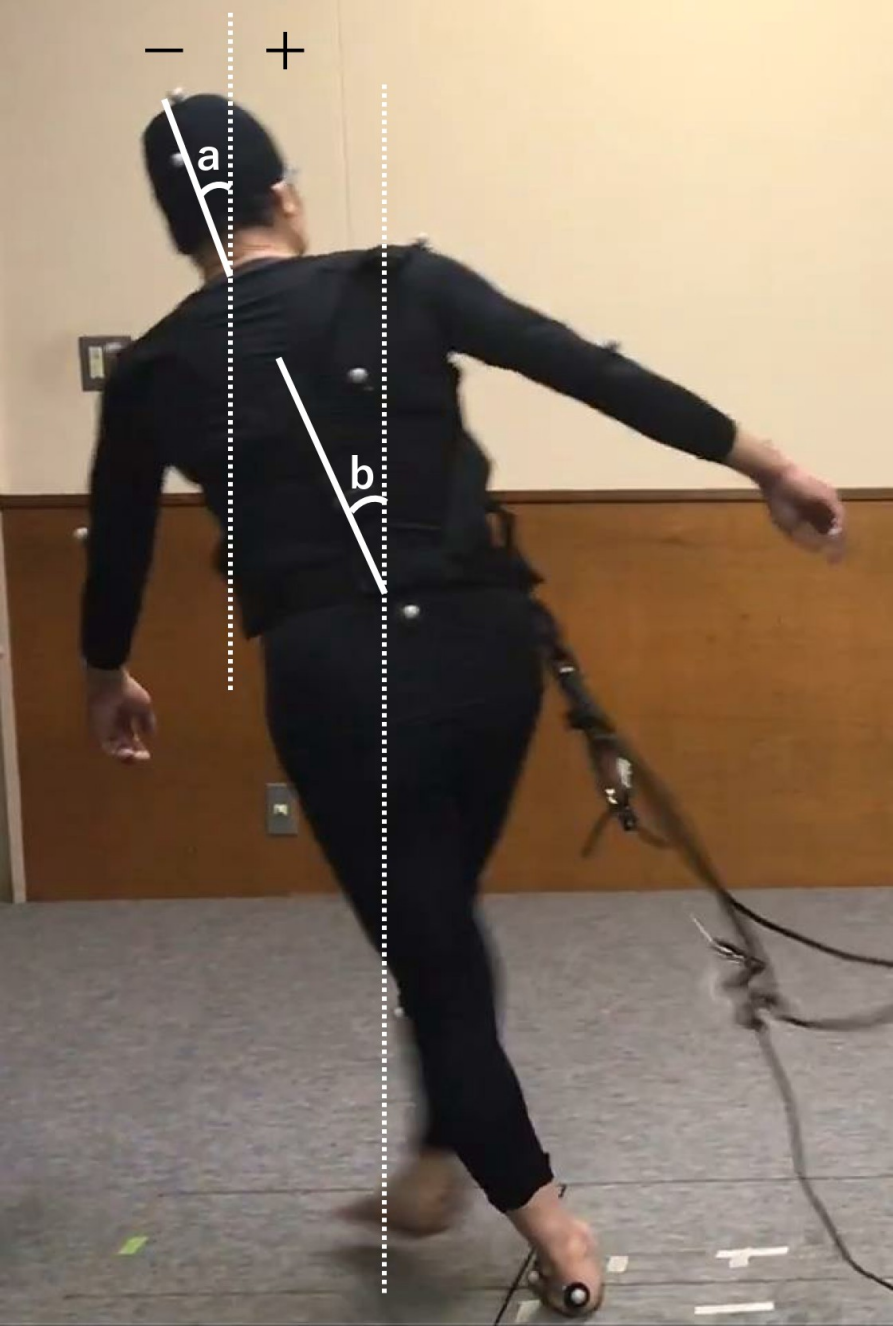
Definition of Head (a) and Trunk (b) Righting Angle Displacement During a Crossover Step.

Motion analysis software (C-motion, Visual3D) was used to analyze the angular displacement, velocity, and moment in the neck and hip joints. Segmental models of the head and torso were created from the camera-derived point data, and the position of each COM was calculated using Winter’s method [12, 13]. The effective acceleration value of the head COM in the analyzed segment was defined as the amount of sway of the head and was calculated for each of the anteroposterior, lateral, and vertical directions. The tilt angle displacement and angular velocity of the head and trunk COM relative to the vertical axis on the frontal plane were calculated and defined as the righting angle displacement and angular velocity, respectively (Fig 2). In addition, the lateral bending moment of the neck and the angular velocity of hip flexion and adduction at the time of step emergence were calculated for the analyzed section, and the maximum value of each parameter was used. The lateral bending moment of the neck was defined by a link segment model with the seventh cervical vertebra as the center of rotation. The neck lateral bending moment was estimated by inverse kinematics calculations using the position and posture of the link model and floor reaction force data estimated by inverse kinematics calculations as input. The positive value of the lateral bending moment of the neck was in the lateral direction, which was opposite to the direction of the external disturbance. The hip angular velocity was calculated for the stepping-leg hip joint, with positive values for forward flexion and leftward adduction (step direction).

Statistical analyses were performed to compare the amount of sway of the head COM in each direction, the tilt angle displacement and angular velocity of the head and trunk COM, and the neck lateral bending moment and hip flexion/adduction angular velocity of the stepping leg between the older and younger groups using Welch’s t-test. In addition, the Pearson correlation coefficient was used to analyze the relationship between the neck lateral bending moment and hip flexion/adduction angular velocity of the stepping leg. The significance level was set at *p* values <0.05.

## Results

For the crossover step, all the participants used the strategy of crossing the stepping leg in front of the supporting leg. In addition, there was no significant correlation between the height of the participants and the righting angle displacement and angular velocity of the head (older group r = 0.32, *p*>0.05, younger group r = 0.03, *p*>0.05).

**Fig 3:**
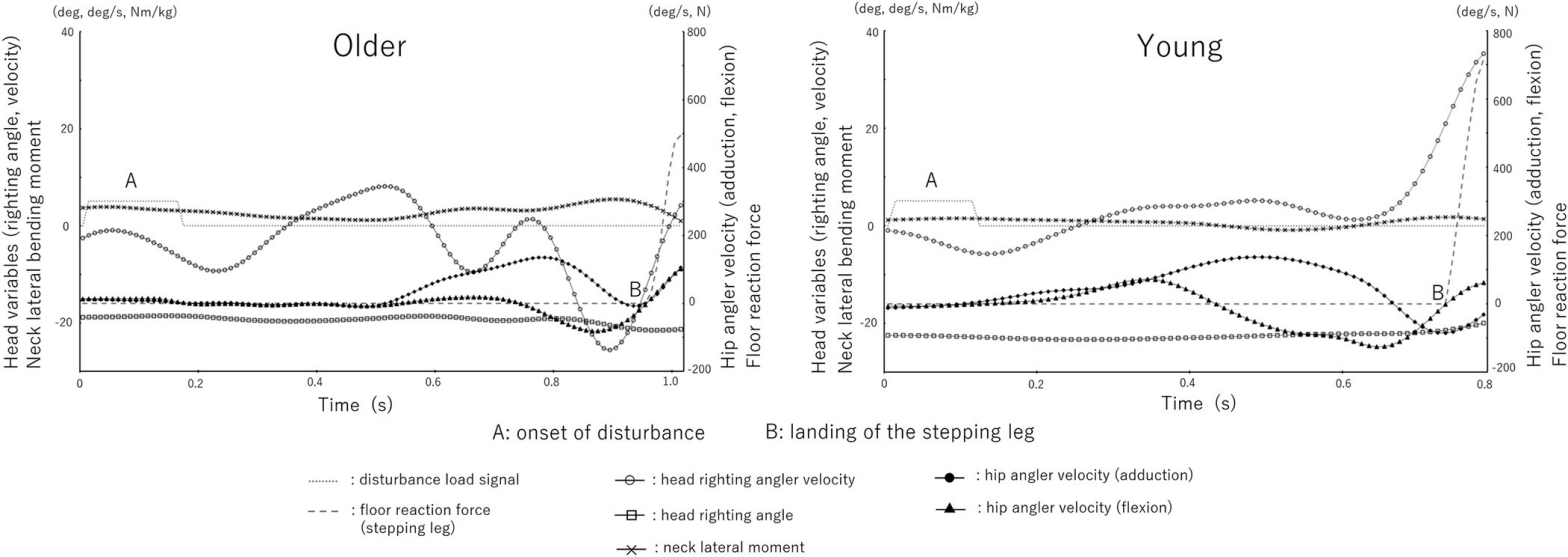
Time course change of the head and hip variables during a representative crossover stepping.

The time course changes of the head and hip variables from disturbance load to the end of the step reaction for a representative participant are shown in Fig 3.

### Sway of the head COM

Table 1 shows the sway of the head COM in each direction during the crossover step. The values were significantly higher in the medial-lateral direction, which is the direction of disturbance, in the older group than in the younger group (*p*<0.05). There were no significant differences between the two groups in the anteroposterior and vertical directions.

**Table 1:** Amount of head swaying, righting angle displacement and angular velocity of the head and trunk, and lateral bending neck moment during crossover steps.

	Head COM sway (m/s^2^)			Righting of the head		Righting of the trunk		Neck lateral bending moment
	A-P	M-L	Vertical	Angle (deg)	Angular velocity (deg/s)	Angle (deg)	Angular velocity (deg/s)	(Nm/kg)
Older	0.94±0.3	1.13±0.7*	0.72±0.3	-14.4±7.1*	9.9±6.6**	2.72±3.9	25.5±12.4	2.5±1.5**
Young	0.92±0.5	0.54±0.3	0.74±0.3	-8.3±4.5	41.2±27.7	3.23±5.1	26.8±20.4	0.7±0.4

A-P, anteroposterior; M-L, medial-lateral; COM, center of mass.

*p<0.05

**p<0.01

### Angle displacement and angular velocity of the head and trunk COM

Table 1 also shows the righting angle displacement and angular velocities of the head during the crossover step. Significantly lower head values were noted for both the righting angle displacement and angular velocity in the older group than in the younger group.

For the trunk, there were no significant differences in the righting angle displacement or angular velocity between the two groups.

### Neck lateral bending moment

Table 1 shows the lateral bending moments of the neck during the crossover step.

Neck lateral bending moments were significantly higher in the older group than in the younger group (p<0.01).

### Angular velocity of flexion and adduction of the stepping leg hip joint

The angular velocity of hip flexion was 39.9±22.8 deg/s in the older group and 18.5±10.7 deg/s in the younger group; that is, this parameter was significantly higher in the older group. The angular velocity of adduction was 206.3±66.7 deg/s in the older group and 202.3±57.4 deg/s in the younger group, with no significant difference between the two groups.

### Relationship between neck lateral bending moment and hip angular velocity

**Fig 4:**
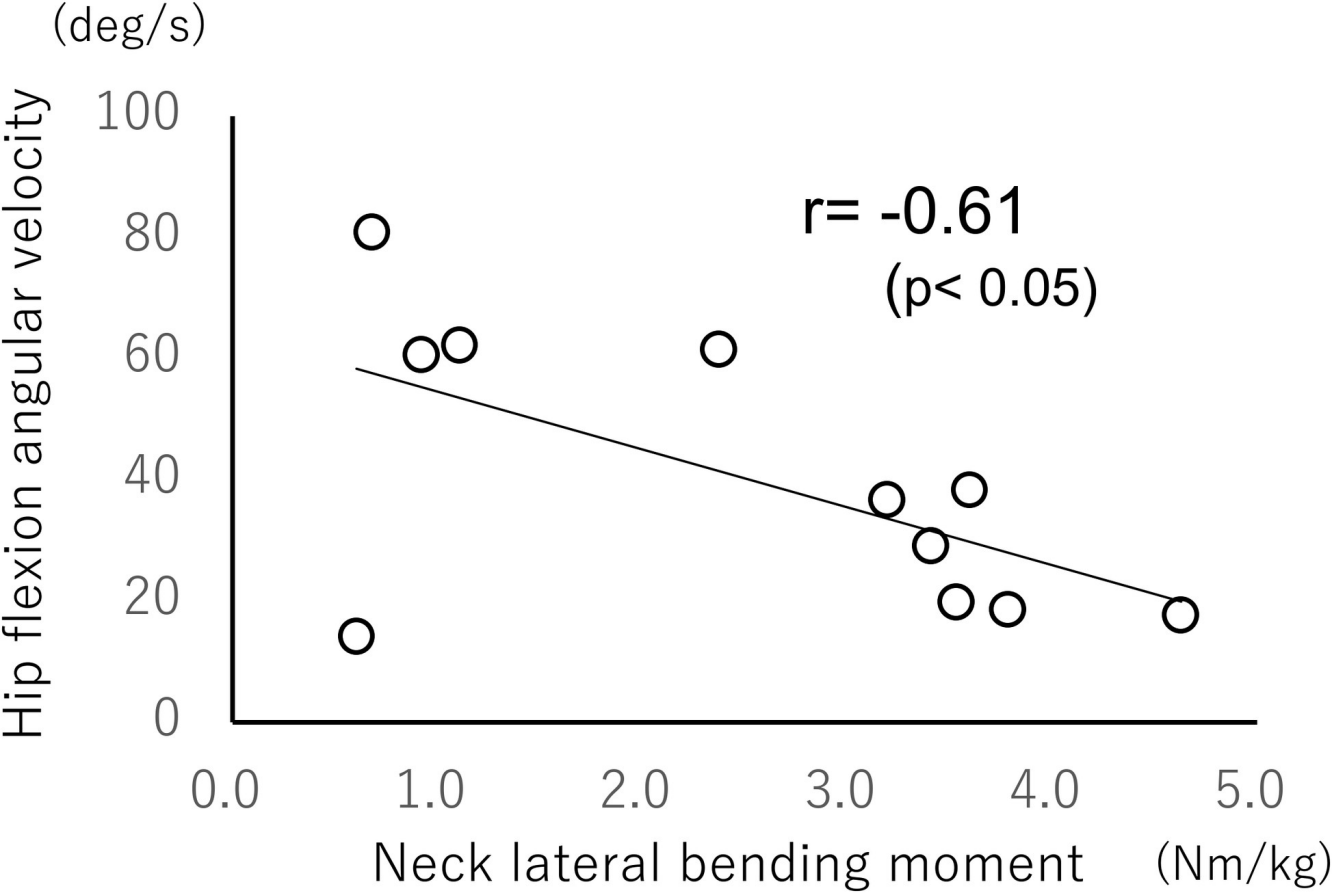
Relationship between neck moment and angular velocity of hip flexion during crossover steps (older group).

**Fig 5:**
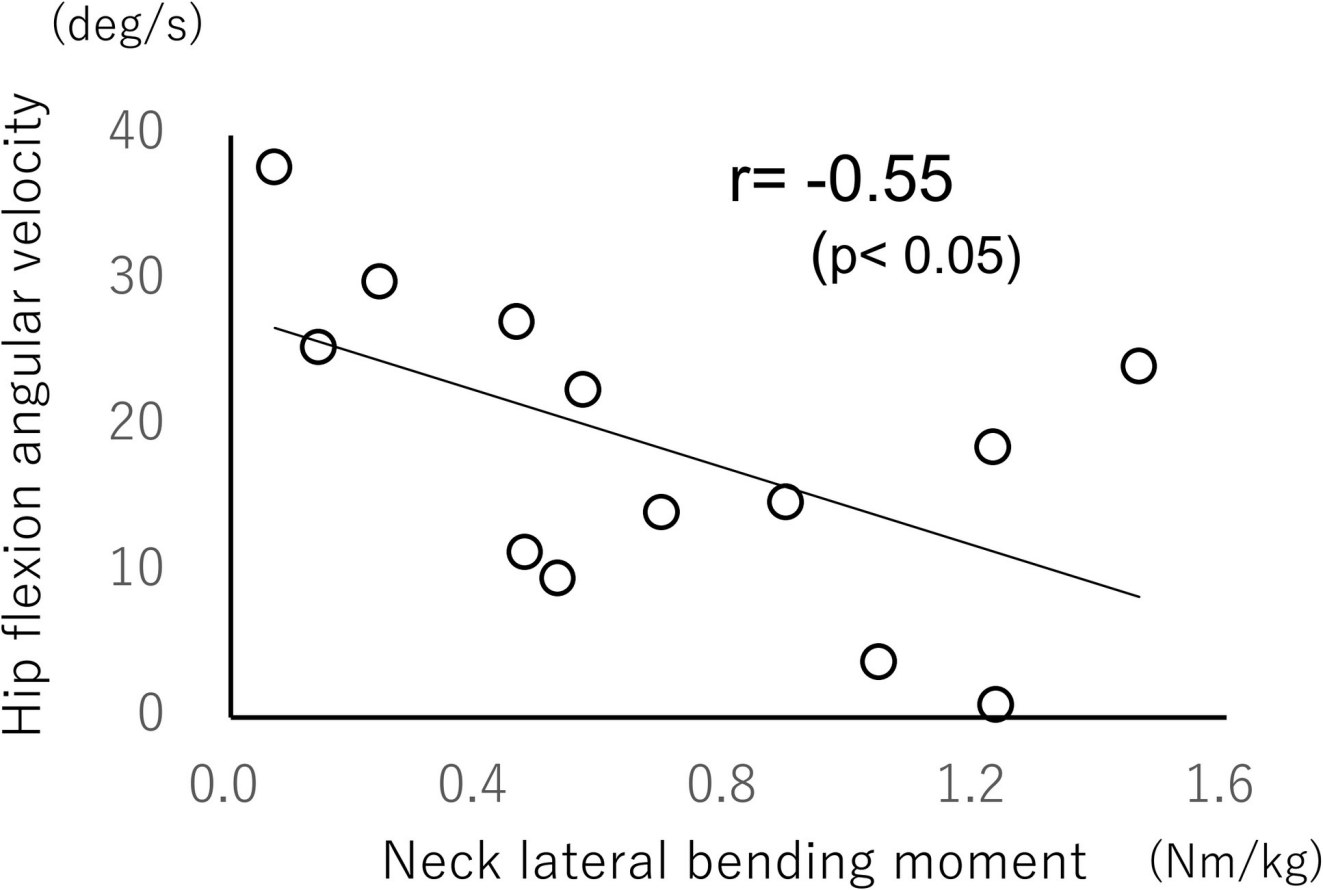
Relationship between neck moment and angular velocity of hip flexion during crossover steps (younger group).

**Fig 6:**
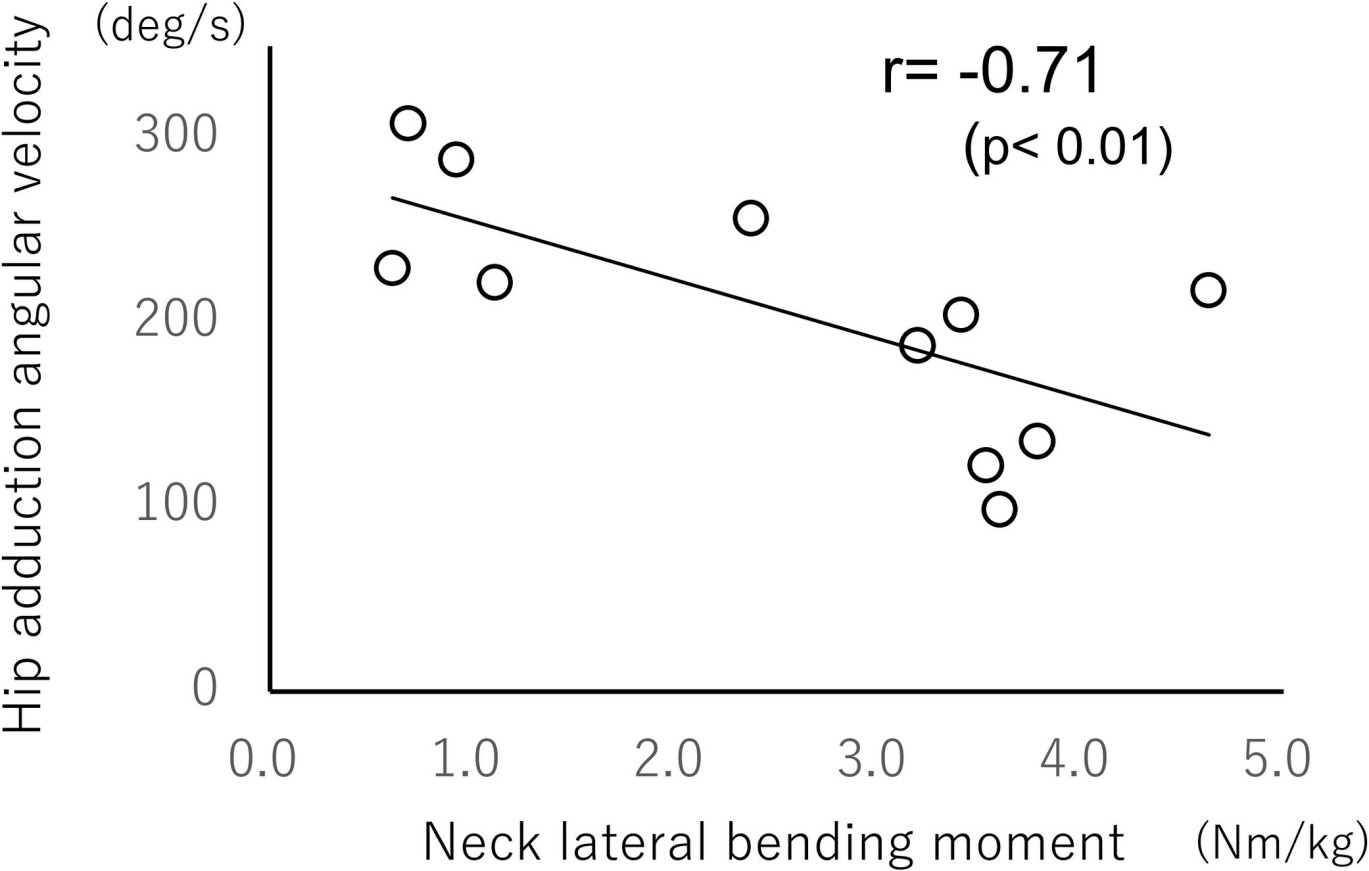
Relationship between neck moment and angular velocity of hip adduction during crossover steps (older group).

Correlation analyses between neck lateral bending moment and hip flexion angular velocity showed significant negative correlations in both older (r = −0.61, *p*<0.05; Fig 4) and younger (r = −0.55, *p*<0.05; Fig 5) groups. Similar correlation analyses of the cervical lateral bending moment and hip adduction angular velocity showed a significant negative correlation only in the older group (r = −0.71, *p*<0.01; Fig 6).

## Discussion

The results of this study confirmed that during the lateral step reaction, the extent of the sway of the head COM was significantly larger in the lateral direction, which was the direction of the disturbance load, in the older group than in the younger group. In this study, the effective acceleration value of the head COM was defined as the amount of sway. This indicates that the ability to control the acceleration of the head in the medial-lateral direction during the lateral step reaction caused by a disturbance was decreased in the older group. Previous studies [10, 11] that investigated the acceleration of the head during step reactions in response to anteroposterior disturbances reported that the linear acceleration and peak angular velocity of the head in the anteroposterior direction was significantly greater in the older group than in the younger group. Moreover, the peak head acceleration used in the previous study [11] was equivalent to the head sway used in this study because it is used to quantify head stability. Age-related degeneration and atrophy of the peripheral vestibular system extend throughout the vestibular apparatus from the otoliths and hair cells to the vestibular nerve [13, 14]. Similar to these findings in the anteroposterior direction, the results of the current study confirm that during the lateral step reaction, the linear acceleration of the head in the lateral direction is greater in older individuals.

In addition, the results for the righting angle displacement and angular velocity of the head COM showed that both parameters were significantly lower in the older group than in the younger group. This indicates that the reaction to an antigravity position, which aims to quickly reestablish the head COM that had been tilted toward the disturbance direction back to a vertical position in space, is reduced during the period from the time of disturbance leading to the landing of the step foot.

The vestibular organ (semicircular canals, as well as saccule and utricle), which contains receptors for equilibrium sensation, senses the acceleration of the head and sends this information to a type of neural integrator called the neural store [15]. In addition to vestibular sensory information, somatosensory information (perception information) and visual information are integrated and processed in the neural store to calculate the appropriate output to the musculoskeletal system. When older individuals lose their balance in the standing posture and recover their balance without falling, the head and trunk remain vertically in space, which reduces the acceleration generated in the vestibular organs and leads to a step reaction in the lower limbs. However, in the case of a fall, a reduced righting of the head leads to greater head acceleration due to increased neck and trunk muscle activity caused by the vestibulospinal reflex. This causes the head and trunk to act as a single mass and tilt, as the head did. As a result, the step reaction of the lower limbs does not occur, and finally, the COM is displaced from the base of the support, leading to a fall [3].

All participants in this study were able to initiate a lateral step reaction (crossover step) in response to the disturbance load. However, the aforementioned results in older individuals showed that the acceleration of the head COM in the medial-lateral direction was significantly increased, whereas both righting angle displacement and angular velocity of the head were decreased. The results suggest that in older adults, greater head acceleration is sensed by the precuneus, which increases cervical muscle activity through the vestibulospinal reflex and decreases head stance angle and angular velocity. In addition, Diehl and Pidcoe reported a significant positive correlation between peak head acceleration and step latency, a measure of head stability [11]. This suggests the possibility of a negative cycle in which reduced righting ability, which reduces head acceleration, leads to reduced regional values of acceleration sensing in the vestibular organs and increased stiffness in the trunk and lower limbs, thereby delaying the onset of the step response.

The results of the neck lateral bending moments during the lateral stepping reaction showed that the older group generated significantly larger moments than the younger group did. This moment is the source of the head righting force in older individuals, in which the neck bends in the lateral direction opposite to the direction in which the disturbance occurs; that is, the head is held in a vertical position in space. The results of this study showed no significant differences between the two groups in terms of the righting angle displacement and angular velocity of the trunk COM. Therefore, the involvement of the trunk in reactive head righting opposite to the direction of disturbance may be small, and greater involvement of the neck lateral bending moment in adjusting the position of the head in space may be a characteristic of older participants. Contrarily, the righting angle displacement and angular velocity values of the head COM opposing the disturbance direction were significantly lower in the older group than in the younger group. In combination with the results of the aforementioned neck lateral direction moment, it is clear that older individuals generated greater rotational forces in the direction opposite to the disturbance direction, but the head righting angle displacement and speed were lower. The lateral bending moment of the neck is used as an index in this study. This internal moment is the product of the muscle contraction force and the distance between the line of the floor reaction force and the seventh cervical vertebra as the center of rotation. Therefore, it reflects the contractility of lateral bending muscles of the neck such as the right sternocleidomastoid and oblique muscles. This suggests that older individuals control the displacement of the head in the lateral direction by strongly contracting the neck lateral bending muscle group in response to the disturbance load. Because the joint moments exerted during lateral neck flexion were calculated and used as an index in this study, the amount of activity and type of contraction of the neck lateral flexor muscle group were not examined; however, the results of this study indicate that although the lateral neck moments were significantly greater in older adults than in younger adults, the head righting angle and angular velocity were lower in older adults. It was inferred that isometric contraction of the neck lateral flexor muscle group in older adults limited the movement of the head in space.

The results of the angular velocity of flexion and adduction of the hip of the supporting leg showed significantly higher flexion values in the older group than in the younger group. In other words, older individuals swung their stepping leg forward faster when a disturbance-induced stepping reaction occurred. Older people often use the side-step sequence in which the lower limb on the side of the disturbance performs a lateral stepping. The crossover step, the task movement in this study, is considered a more difficult reaction than the side-step sequence because the stepping leg crosses the front of the supporting leg [3, 7, 8]. The results of the present study suggest that in older individuals, the angular velocity of hip flexion was increased at the onset to avoid contact between the stepping leg and supporting legs.

The results of the correlation analyses between the neck lateral bending moment and hip flexion angular velocity showed a significant negative correlation in both groups. This indicates that the participants who demonstrated greater neck lateral bending moments had lower hip flexion angular velocities. Considering the aforementioned theory [3] of the relationship between neck muscle activity and lower limb stiffness, a stronger neck lateral bending moment or muscle contraction of the cervical lateral bending muscle group causes the head and trunk to become one fixed mass, thereby increasing head acceleration. As an effect of aging, the vestibular organ senses the stronger head acceleration, which increases the muscle activities and stiffness of the lower limbs, decreases the angular velocity of flexion of the stepping leg hip joint during disturbance loading, and further decreases the angular velocity of adduction to generate a new base of support in the direction of disturbance loading.

The results of this study indicate the need to evaluate the angle/angular velocity of the head in the frontal plane in combination with the kinematics of the lower extremities during the crossover step. Evaluation of head kinematics during the step movement would allow for the planning and implementation of highly individualized fall prevention training for older adults, including training content for the vestibular sensory system. One limitation of this study is that we did not examine the participant’s physical characteristics, such as lower extremity muscle strength and walking ability, which are involved in the compensatory step reaction, or history of falls. In addition, although this study analyzed kinematic characteristics mainly on the frontal plane, crossover step movements may involve movements on the horizontal plane, such as rotational movements of the trunk and neck. Furthermore, several previous studies have linked step response to cognitive function that declines with age [16–18]. We believe that investigating these factors in combination in the future will enable us to investigate the details of crossover step movements in older people.

## Conclusions

The results of the present study indicate that during crossover stepping in response to external disturbances, the lateral instability of the head increases with aging, the head righting decreases, and the head and trunk become one mass as the muscle activities increase to exert the neck lateral bending moment, resulting in increased head acceleration. Our data suggest that the increased stiffness of the trunk and lower extremities due to vestibulospinal reflexes associated with increased head acceleration reduces the angular velocity of hip flexion and adduction of the stepping leg in crossover stepping.

Future research should collect data on changes in head control ability and step response in older adults before and after vestibular physiotherapy, which has been shown to be effective in improving posture and gait stability [19].

## Supporting information

S1 Data Means and standard deviations. (PDF)

S2 Data Data for graphs (representative values). (XLSX)
