# Supplementary material for: Influence of aging on the relation between head control and hip joint kinematics during crossover stepping
Source: PLoS One. 2024 May 24;19(5):e0299850. doi: 10.1371/journal.pone.0299850 (PMC11125510; doi:10.1371/journal.pone.0299850)
Supplement: S1 Data — (PDF) [file pone.0299850.s001.pdf]

S1 Table Means and standard deviations for each parameter in the older and younger groups

	subject	Head COM sway			Righting of the head		Righting of the Trunk		neck_moment	Hip joint angular velocity	
		AP	ML	Vertical	Angle	Angular velocity	Angle	Angular velocity		flexion	adduction
		(m/s^2)	(m/s^2)	(m/s^2)	(deg)	(deg/s)	(deg)	(deg/s)	(Nm/Kg)	(deg/s)	(deg/s)
Older	O1	1.34	2.78	0.57	-20.77	2.06	8.24	21.77	3.41	29.02	204.07
	O2	0.65	1.88	0.99	-15.34	3.89	3.87	24.25	4.63	17.62	217.43
	O3	1.39	0.58	0.53	-6.17	19.78	7.64	35.20	3.53	19.78	122.44
	O4	0.76	1.63	1.14	-17.51	17.13	0.20	32.80	2.37	61.46	256.46
	O5	0.73	0.49	0.66	-6.17	2.15	5.11	33.24	1.11	62.17	221.71
	O6	0.94	0.49	0.33	-28.55	7.93	-0.66	10.73	3.60	38.30	98.68
	O7	1.12	0.64	0.97	-18.54	8.15	-3.63	14.15	3.79	18.54	135.63
	O8	1.14	1.38	1.21	-14.81	14.75	-1.38	44.59	0.68	80.86	307.94
	O9	0.58	0.41	0.45	-6.07	8.61	2.49	2.18	3.19	36.63	187.49
	O10	1.09	1.15	0.59	-14.72	5.87	1.83	28.65	0.61	14.11	229.56
	O11	0.63	0.94	0.48	-9.31	18.77	6.17	32.69	0.92	60.58	288.40
	mean	0.94	1.12	0.72	-14.36	9.92	2.72	25.48	2.53	39.92	206.35
	sd	0.29	0.74	0.30	7.07	6.59	3.86	12.41	1.45	22.85	66.68

	subject	Head COM sway			Righting of the head		Righting of the Trunk		neck_moment	Hip joint angular velocity	
		AP	ML	Vertical	Angle	Angular velocity	Angle	Angular velocity		flexion	adduction
		(m/s^2)	(m/s^2)	(m/s^2)	(deg)	(deg/s)	(deg)	(deg/s)	(Nm/Kg)	(deg/s)	(deg/s)
Young	Y1	0.59	0.45	0.52	-3.80	12.87	5.07	1.63	1.23	0.89	206.92
	Y2	1.71	0.45	0.31	-7.72	9.69	-3.48	52.13	0.57	22.51	228.25
	Y3	0.99	0.60	0.44	-5.19	65.66	2.74	7.53	0.46	27.18	52.79
	Y4	1.06	0.46	0.45	-10.43	41.97	-2.92	43.34	1.46	24.14	194.10
	Y5	0.63	0.66	0.70	-16.35	28.38	3.24	3.04	0.89	14.79	184.74
	Y6	2.20	0.35	1.01	-2.00	90.47	-4.19	49.86	1.04	3.82	227.29
	Y7	0.66	0.79	1.26	-8.73	38.92	11.17	14.77	1.23	18.56	222.72
	Y8	0.43	0.28	1.16	-8.03	37.90	7.96	47.47	0.47	11.37	297.70
	Y9	0.60	0.62	0.55	-4.56	16.78	7.14	52.69	0.14	25.44	230.45
	Y10	1.32	0.35	0.68	-14.64	58.92	5.08	25.62	0.53	9.55	195.53
	Y11	0.52	0.70	0.59	-12.04	25.27	5.51	32.78	0.24	29.95	148.54
	Y12	0.78	1.13	1.51	-3.30	90.89	-2.93	5.30	0.69	14.09	248.74
	Y13	0.50	0.14	0.49	-10.61	17.66	7.64	11.85	0.07	37.80	192.24
	mean	0.92	0.54	0.74	-8.26	41.18	3.23	26.77	0.69	18.47	202.31
	sd	0.53	0.26	0.37	4.45	27.67	5.07	20.37	0.44	10.66	57.41
